# Cerebral Perfusion Monitoring Using Near-Infrared Spectroscopy During Head-Up Tilt Table Test in Patients With Orthostatic Intolerance

**DOI:** 10.3389/fnhum.2019.00055

**Published:** 2019-02-19

**Authors:** Yoo Hwan Kim, Seung-ho Paik, Zephaniah Phillips V, Nam-Joon Jeon, Byung-Jo Kim, Beop-Min Kim

**Affiliations:** ^1^Department of Neurology, Hallym University College of Medicine, Seoul, South Korea; ^2^Department of Neurology, Korea University Medical Center, Seoul, South Korea; ^3^Department of Bio-convergence Engineering, Korea University College of Health Science, Seoul, South Korea; ^4^Neurophysiology Laboratory, Korea University Anam Hospital, Seoul, South Korea; ^5^Brain Convergence Research Center, Korea University Anam Hospital, Seoul, South Korea

**Keywords:** orthostatic intolerance, near-infrared spectroscopy, tilt table test, cerebral blood flow, hemodynamics

## Abstract

The head-up tilt table test (HUT) is one of the primary clinical examinations for evaluating orthostatic intolerance (OI). HUT can be divided into three phases: dynamic tilt phase (supine to tilt up), static tilt phase (remain tilted at 70°), and post tilt phase (tilt down back to supine position). Commonly, blood pressure (BP) and heart rate (HR) are monitored to observe for OI symptoms, but are indirect measurements of cerebral perfusion and can lead to inaccurate HUT evaluation. In this study, we implemented a 108-channel near-infrared spectroscopy (NIRS) probe to characterize HUT performance by monitoring cerebral hemodynamic changes for healthy controls (HCs), OI patients with normal HUT results, and OI patients with positive HUT results: vasovagal syncope (VS), postural orthostatic tachycardia syndrome (POTS), orthostatic hypotension (OH), and orthostatic hypertension (OHT). By the end of the static tilt phase, OI patients typically did not show a complete recovery back to baseline cerebral oxygenation and total blood volume compared to HCs. We characterized the return to cerebral homeostasis by polynomial fitting total blood volume changes and determining the inflection point. The OI patients with normal HUT results, VS, OH, or OHT showed a delay in the return to cerebral homeostasis compared to the HC group during HUT.

## Introduction

The autonomic nervous system (ANS) is essential for maintaining homeostasis in response to environmental changes, for example, maintaining cerebral hemodynamics by regulating blood pressure (BP) and heart rate (HR). However, when the ANS is disturbed, various symptoms related to ANS dysregulation, such as dizziness, lightheadedness, blurred vision and palpitation are observed, including more severe symptoms such as fainting. The head-up tilt table test (HUT) is a validated, quantifiable autonomic function test that is widely used as a diagnostic tool to evaluate patients with orthostatic intolerance (OI). The HUT was initially introduced to diagnose vasovagal syncope (VS; Kenny et al., 1986). The use of the HUT has since expanded to demonstrate physiological events mediated by transient cerebral hypo-perfusion.

One of the major problems with the HUT is the high false-positive and false-negative rates in adults (Wieling et al., 2004). This might be due to the fact that that changes in BP and HR, which are two variables normally monitored during HUT, may occur after the point of declining cerebral perfusion (Szufladowicz et al., 2004). The lack of cerebral perfusion monitoring may be one of the reasons for the HUT’s inability to detect impending OI symptoms especially syncope. In fact, the HUT can determine the cause of syncope in only 40%–80% of cases. Moreover, 10%–30% of healthy patients with no history of syncope have an abnormal HUT result (Low, 1997; Jones and Gibbons, 2015).

Since OI symptoms are not well correlated with orthostatic BP or HR, there is a limit to assessing OI using traditional autonomic function testing alone. We propose using near-infrared spectroscopy (NIRS) to measure changes in cerebral perfusion and blood oxygenation during the HUT in healthy controls (HCs) and in patients with suspected OI. Patients with OI have been shown to have reduced cerebral perfusion and blood oxygenation during the HUT. Bachus et al. (2018) have reported cerebral deoxygenation during the HUT for patients with VS and have concluded that NIRS was a valuable method for systematic investigation of OI patients. However, this study focused mainly on VS and general OI symptoms (Bachus et al., 2018). Other studies of NIRS measurements during the HUT either did not have a diverse group of patient or utilized a small number of NIRS channels (Ayers and Lawrence, 2015). In this study, we monitored the hemodynamic changes during the three phases of the HUT: dynamic tilt phase (supine to tilt up), static tilt phase (remain tilted at 70°) and post tilt phase (tilt down back to supine position) and compare the results between HCs and various OI patient groups. Our customized 108-channel NIRS system was utilized to measure the relative changes of oxyhemoglobin (HbO), deoxyhemoglobin (Hb), and total hemoglobin (HbT). We aimed to demonstrate NIRS as a viable tool for monitoring cerebral perfusion changes during the HUT for various OI patient groups.

## Materials and Methods

### Subjects

Patients with symptoms of OI were recruited based on the following criteria: (1) symptoms suggesting OI (memory loss, visual difficulties, lightheadedness, headache, fatigue, increases or decreases in BP, weakness, nausea and abdominal pain, sweating, tremulousness, and exercise intolerance (Low et al., 1995); (2) no history of central nervous system disease (e.g., stroke, Parkinson’s disease, or Alzheimer’s dementia); and (3) no history of significant head injury, alcohol, or psychotropic drug abuse.

Demographic and clinical data including age, sex, and comorbid chronic diseases were also obtained. Healthy control volunteers who did not have any medical history that might affect ANS were recruited. In addition, subjects with severe arrhythmia were excluded due to the difficulty of a HUT analysis. All participants gave written informed consent before study inclusion. All procedures were performed in accordance with the Declaration of Helsinki and were approved by the Korea University Medical Center Institutional Review Board.

### Study Design

Subjects were selected if they most clearly showed changes in BP and HR pertaining to each subject group, along with usable NIRS data. Knowing that the ANS may be influenced by changes in the environment, psychological state, and medication (Shields, 2000) and to minimize the effects of these confounders, all participants taking medications that affect autonomic function were asked to discontinue their medicine for at least 24 h prior to the exam. The HUT was controlled and regulated by the standard electrodiagnostic laboratory environment (Koo et al., 2012; Kim et al., 2014a,b). Tests were performed in the following sequence: (1) the patient completed an autonomic dysfunction self-questionnaire; and (2) the HUT was conducted after attaching the NIRS probe on the forehead to monitor cerebral hemodynamics.

### Autonomic Dysfunction Questionnaire

The self-rated questionnaire was employed to assess the severity of autonomic dysfunction. Autonomic dysfunction was measured using the Korean version of the Composite Autonomic Symptom Score 31 (K-COMPASS 31) consisting of 31 items that assessed six different domains: four items for OI, three items for vasomotor, four items for secretomotor, 12 items for gastrointestinal, three items for bladder, and five items for pupillomotor. The sum of the items from each domain and the sum of all 31 items were used for analysis. Of note, higher scores indicate a more severe autonomic dysfunction (Sletten et al., 2012). The minimum total score of K-COMPASS 31 is 0 and the maximum score is 100. We also scored only the four items related to OI separately among the many items of the K-COMPASS 31 questionnaire with the sum of these four items ranging from 0 to 10.

### The HUT Protocol

The HUT was performed using Finometer equipment (Finapres Medical Systems BV, Amsterdam, Netherlands) with a cuff placed on the middle finger and a sphygmomanometer cuff simultaneously placed over the brachial artery serial measurements of BP (systolic, diastolic, and mean BP) as well as HR were performed. Systolic and diastolic BP were displayed on a monitor console. After a 20-min rest in the supine position, baseline BP and HR were recorded. Then, each subject was slowly positioned to an angle of 70° on a standard electrically driven tilt table with a footboard. The time to tilt, from supine to 70°, and vice versa, depends on the weight of the subject but was approximately 20 s. In fact, 20 s is a few seconds longer than normal tilting speeds of 5°/s (Sundkvist and Lilja, 1983; Jahan et al., 1996) mostly because of the careful consideration of the NIRS system attached to the subject and tilt bed. The subject remains tilted for up to 10 min (Novak, 2011) and for some exceptions, the test was performed for up to 30 min (e.g., in case of mild OI). The test was stopped early if the OI symptoms occurred during the test. Resting-state BP and HR were measured 10 min after returning the table to the supine position. The data were used for analysis only if BP and HR values measured by the two instruments matched. Pharmacologic provocation was not performed.

### Patient Groupings

Based on the response pattern of the HUT, OI patients were grouped as abnormal OI (AOI) [abnormal head-up tilt table test results due to VS, postural orthostatic tachycardia syndrome (POTS), orthostatic hypotension (OH), or orthostatic hypertension (OHT)] or NOI (normal head-up tilt table test results despite OI). We compared these two patient groups with HCs who had no OI and whose HUT was normal. The AOI diagnoses were defined as follows. Patients were diagnosed with OH if a reduction in systolic BP of at least 20 mmHg or a reduction in diastolic BP of at least 10 mmHg was recorded within 3 min of standing up Low and Tomalia (2015). Patients were diagnosed with OHT if a postural increase in systolic BP by at least 20 mmHg was recorded (Fessel and Robertson, 2006; Novak, 2016). Patients who displayed HR increases of more than 30 beats per min (bpm) or maximum HR ≥120 bpm within the first 10 min without evidence of OH were diagnosed as POTS (Freeman et al., 2011). In young children, a higher HR threshold (≥40 bpm) was used, as younger children have a greater orthostatic tachycardia (Raj, 2013). Patients were diagnosed with VS if they exhibited spontaneous syncope associated with hypotension, bradycardia, or both (Novak, 2016). As soon as syncope symptoms occurred, patients were returned quickly to the supine position. We defined NOI as a group that has symptoms of OI but does not exceed a 10% change in BP and HR relative to baseline; hence the normal HUT results. Our screening was strict, so subjects who showed any deviation from the clinical definitions listed above or healthy criteria were omitted from the study. This limited our subject pool, but gave us the most qualified participants to present the typical BP, HR, and NIRS response in each group.

### NIRS

A custom-built, 108-channel NIRS probe with dimensions of 120 mm by 40 mm was used to monitor the subject’s entire prefrontal HbO, Hb, and HbT changes during the HUT. The NIRS probe has 12 sources and 15 detectors, for a combination of 108 channels. The 108 channels consisted of 40 channels of 15 mm source-detector (SD) distance, 20 channels of 30 mm SD distance, 32 channels of 36 mm SD distance, and 16 channels of 45 mm SD distance, with the longer channels having deeper penetration into the prefrontal cortex. From our experience, and as shown previously, 30 mm and 36 mm channels have the best tradeoff between noise levels while also being sensitive to cerebral changes (Strangman et al., 2013). Good channel contact between the skin and hardware is ensured by real-time monitoring of to check for saturation or low intensity levels. The experiment administrator instructed the subjects to avoid large head motions that may induce motion artifacts in the NIRS signal. Although the NIRS probe had bluetooth capability, the system was powered and transmitted data through a laptop in the room. For removing additional noise and motion artifacts, a wavelet-based de-noising method (Daubechies 5) was applied to the NIRS signals. This method has been shown to be effective in removing sudden and drastic changes in time-series data induced by motion artifacts (Molavi and Dumont, 2012). A more detailed review of the NIRS methodology, principles, and terminology can be found in our previous article (Kim et al., 2018).

### Statistical Analysis

Differences in demographic characteristics, questionnaire scores, and proportion of subjects with abnormal results from the HUT among the patient groups and the HC group were analyzed using either the Kruskal–Wallis test or Fisher’s exact test. The NIRS parameters were calculated and compared between the five OI patient groups and HC subjects using custom-built scripts written in MATLAB (2010A, The Math Works Inc., Natick, MA, USA). NIRS data was analyzed using a two-tailed *t*-test to determine statistical significance (*p*-value < 0.05).

## Results

### Subject Characteristics

A total of 38 patients with OI and 12 healthy subjects were recruited. However, four patients with OI and two healthy subjects were excluded from analysis because they showed severe arrhythmia which made BP/HR parameters unreliable. Two additional healthy subjects were also excluded because of excessive motion artifacts in the NIRS signal and a lack of good SD contact, respectively, during the HUT.

Finally, data from 34 patients with OI and eight healthy subjects were analyzed. The patients with OI symptoms were grouped according to the findings of the HUT as follows: (1) VS group (*n* = 4; median age 30.0; one male); (2) POTS group (*n* = 2; median age 31.0; one male); (3) OH group (*n* = 7; median age 72.0; four males); (4) OHT group (*n* = 5; median age 66.0; one male); (5) NOI group (*n* = 16; median age 54.0; five males); and (6) HC group (*n* = 8; median age 67.5; five males). Table 1 presents the demographic and clinical characteristics including the HUT results of all subject groups.

**Table 1:** Demographic and clinical characteristics of subjects.

	*Group* (*n* = 42)						
	VS (*n* = 4)	POTS (*n* = 2)	OH (*n* = 7)	OHT (*n* = 5)	NOI (*n* = 16)	HC (*n* = 8)	*p*-value
*Clinical features*							
Sex ratio (M:F)	1:3	1:1	4:3	1:4	5:11	5:3	0.503
Age (*y*)	30.0 (23–72)	31.0 (20–42)	72.0 (42–83)	66.0 (38–73)	54.0 (22–79)	67.5 (57–74)	0.054
Height (m)	1.65 (1.61–1.75)	1.71 (1.69–1.72)	1.60 (1.42–1.74)	1.57 (1.48–1.70)	1.63 (1.49–1.73)	1.63 (1.54–1.77)	0.323
Weight (kg)	58.5 (48–64)	61.5 (60–63)	63.0 (50–71)	55.0 (47–65)	62.0 (43–84)	61.0 (44–71)	0.604
BMI (m^2^/kg)	20.3 (23.2–18.5)	21.2 (21.0–21.3)	24.6 (22.2–26.3)	22.8 (16.3–29.7)	23.3 (16.8–29.7)	21.5 (18.3–25.5)	0.243
*Questionnaires*							
K-COMPASS 31	16.0 (5–19)	10.0 (8–12)	18.0 (7–37)	9.0 (2–14)	19.5 (6,29)	2.5 (0,11)	**0.001**
OI score	6.0 (2–7)	2.0 (1–4)	4.0 (1–8)	2.0 (1–6)	4.0 (2,9)	0.00 (0,0)	**0.028**

*Data are presented as median (range; minimum, maximum). OH, orthostatic hypotension; OHT, orthostatic hypertension; POTS, postural orthostatic tachycardia syndrome; VS, vasovagal syncope; NOI, normal head-up tilt table test results with OI; HC, healthy control; BMI, body mass index; OI, orthostatic intolerance. Note: Statistically significant values (*p* < 0.05) are indicated in bold*.

### NIRS Response to the HUT

**Figure 1:**
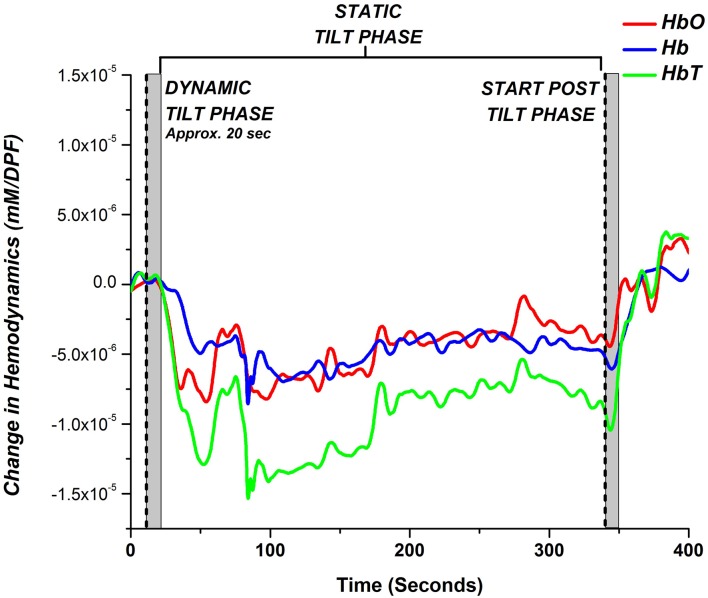
Typical hemodynamic response of a healthy control (HC) during a head-up tilt table test (HUT) examination. The hemodynamic response for the HUT can be partitioned into three distinct sections: dynamic tilt phase (supine to tilt up), static tilt phase (remain tilted at 70°), and post tilt phase (tilted down back to supine position). The gray bar marks the approximate mechanical tilting time (approximately 20 s, depending on weight).

The NIRS response to the HUT can be divided into three phases: dynamic tilt phase, static tilt phase, and post tilt phase, as shown in Figure 1 for a HC subject. The experimental moderator marked in the NIRS software (indicated by the dashed lines in Figure 1) when the table begins to tilt up (start of the dynamic tilt phase) and when it begins to tilt down (start of the post tilt phase). The phase preceding the start of the dynamic tilt phase is considered as baseline. As mentioned previously, tilting is a mechanically driven process that took approximately 20 s. The duration of the static phase was different for each subject and took as much time as needed in order to observe BP/HR recovery or OI symptoms. Therefore, a time-averaged value across all subjects could not be performed for NIRS trends analysis. We divided the NIRS data according to the three HUT phases and compared the hemodynamic response (HbO, Hb, and HbT) during these phases. Due to our limited but qualified subject pool, our aim was to observe whether NIRS monitoring during the three HUT phases added any potential benefit to traditional BP/HR monitoring.

**Figure 2:**
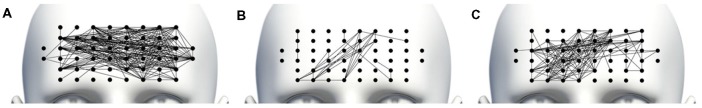
Channel correlation density map for **(A)** HC, **(B)** normal OI (NOI) and **(C)** abnormal OI (AOI) subjects. A line was drawn connecting channel pairs whose average correlation coefficient was greater than or equal to 0.8.

We investigated if there were any regional dependencies for blood volume changes over the prefrontal areas for the HC, NOI, and AOI groups. For each subject, we calculated the correlation coefficient for HbT changes during the entire time course of the HUT examination, between all 30 mm and 36 mm channel pairs. We then calculated the average channel correlation coefficient between all subjects within each group. Figure 2 displays channel correlation density maps on the prefrontal area for HC (*n* = 8), NOI (*n* = 16), and AOI (*n* = 18) groups. To construct the channel correlation density map, a line is drawn between channel pairs whose average correlation coefficient values were greater than or equal to a threshold of 0.8, indicating a strongly positive correlation between channel time series changes. As depicted in Figure 2A, HCs showed a high amount of correlated channel pairs across the prefrontal area, indicating a lack of regional dependency. The AOI group also showed a high number of channel pairs that meet the threshold, but the channels are slightly biased towards the right side. On the other hand, the NOI group showed the least amount of channels pair which met the threshold, with most channel pairs being located near the center of the prefrontal area. With that being said, each group showed a moderate to strong correlation coefficient for all channel pairs. The HC, NOI, and AOI group’s average correlation coefficient for all channel pairs was 0.62 ± 0.21, 0.44 ± 0.22, and 0.57 ± 0.21, respectively. Therefore, to minimize the influence of noise in individual channels and to generally characterize the hemodynamic response changes for HC, NOI, and AOI groups during the HUT, further analysis was performed by averaging all 30 mm and 36 mm channels.

**Figure 3:**
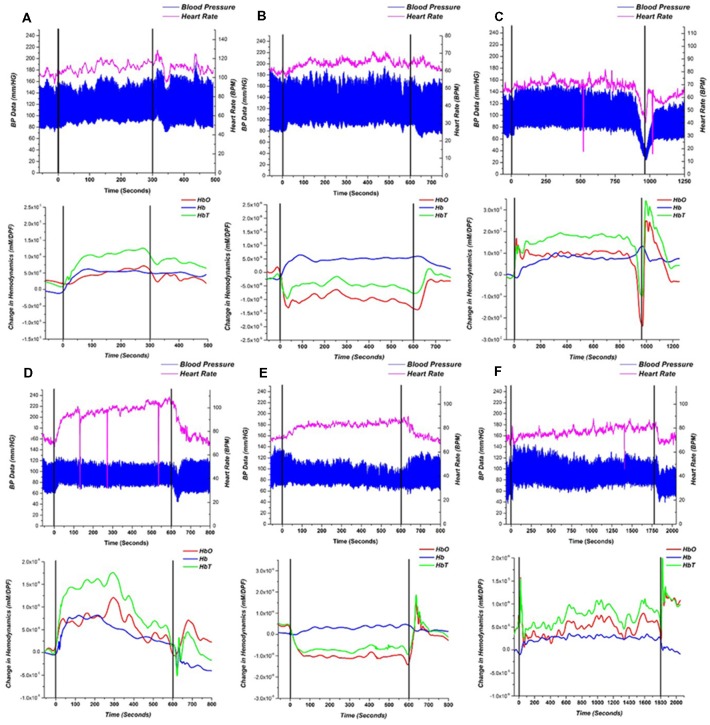
Representative recordings of blood pressure (BP)/heart rate (HR; top) and near-infrared spectroscopy (NIRS; bottom) responses for **(A)** HC, **(B)** NOI patient and **(C–F)** AOI patient with: **(C)** vasovagal syncope (VS), **(D)** postural orthostatic tachycardia syndrome (POTS), **(E)** orthostatic hypotension (OH) and **(F)** orthostatic hypertension (OHT). Starting points of dynamic tilt phase and post tilt phase are shown as black lines. BP is represented as a high low (systolic/diastolic) chart in blue and the HR is represented as the magenta line. The NIRS response is shown as HbO—red, Hb —blue, HbT—green.

A single-subject representative case of NIRS, BP, and HR response to the HUT for a HC, NOI, and AOI groups can be seen in Figure 3. Referring only to the BP and HR measurements, both the HC subject (Figure 3A) and NOI patient (Figure 3B) had stable BP and HR data throughout the three phases of the HUT. However, when we investigated their NIRS response, we saw that the HC subject’s hemodynamics recovered back to baseline by the end of the static tilt phase, while the NOI patient’s HbO and HbT remained below baseline levels, indicating a lack of hemodynamic recovery by the end of the static tilt to post tilt phase.

As mentioned earlier, the AOI group was divided into four groups based on the following four different diagnoses: VS (*n* = 4), POTS (*n* = 2), OH (*n* = 7), and OHT (*n* = 5). Figures 3C–F show a single representative case for each diagnosis within the AOI group. For the VS patient (Figure 3C), when syncope occurred, HbO and HbT fell in a similar pattern to the BP and HR changes, but Hb increased at the start of the syncope event, similar to prior studies. (Bachus et al., 2018) In the POTS patient (Figure 3D), BP was stable during the static tilt phase but HR increased dramatically at the beginning of the static tilt phase. Concentrations of HbO, Hb and HbT suddenly increased, potentially mediated by the increasing HR, and then began to fall in the middle of the static phase only to show an HbO and HbT overshoot when entering the post tilt phase. The OH (Figure 3E) patient conformed to the definition of typical OH, whereas baseline data should be stable in supine position before the dynamic tilt phase. From the dynamic tilt to static tilt phase, BP decreased and HR slightly increased (≤10 BPM). In the NIRS analysis, HbO and HbT falls in the same pattern as the BP data and remains reduced throughout the static tilt phase. In the post tilt phase, i.e in the supine position, the concentration of HbO and HbT recovered back to baseline levels matching the trend in the BP data. For the OHT case (Figure 3F), BP increased within 3 min of the static tilt phase and gradually decreased to baseline. HR shows a gradual increase during the beginning of the static tilt phase. The concentration of HbO and HbT in the OHT case decreased temporarily in the early stages of the static tilt phase, but unlike those seen in the OH case, the decline was not large, and the concentration gradually recovered and rose above baseline. Note that the sharp drops in HR data, seen especially in Figures 3C,D,F, can be attributed to hardware issues in which data was missing for that time point.

**Figure 4:**
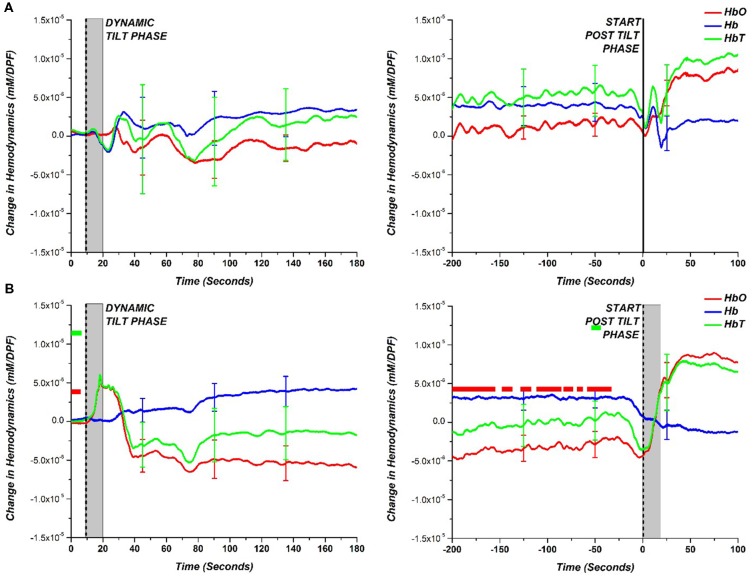
Comparison of hemodynamic response (HbO—red; Hb—blue; HbT—green) during the dynamic tilt to the static tilt phase (left) and the end of static tilt to the post tilt phase (right) for **(A)** HC and **(B)** NOI. The gray bar marks the approximate mechanical tilting time (approximately 20 s, depending on weight). Statistically significant difference (*p*-value < 0.05) between NOI patients and HC is denoted with a square colored to match the respective hemodynamic response. The appearance of a bar implies a long time interval of statistically significant points.

A more general hemodynamic trend of the HC and NOI patient groups during the HUT is depicted in Figure 4 which shows the comparison of the average group hemodynamic response (with standard error bars) for the dynamic tilt to static tilt phase and end of the static tilt to post tilt phase. At each data point for NOI, a *t*-test between HC and NOI subjects was conducted to determine statistical significance (*p*-value < 0.05). The main effect of the group was not tested before conducting *t*-tests as we just looked at a statistically significant difference in means. If a difference was found, a colored square was placed at that time point with the color matching the respective hemodynamic response (i.e., red square for HbO). The appearance of a bar on the figure implies a series of statistically significant points.

For the dynamic tilt to static tilt phase, there was no statistically significant difference among data points between HC and NOI patients. However, by the end of the static tilt phase to post-tilt phase, the HC hemodynamics returned to baseline, matching the starting dynamic tilt phase levels. In the case of NOI, by the end of the static tilt phase, HbO did not return to baseline levels seen at the start of the dynamic tilt phase. The NOI’s HbO data was significantly different from that of HCs for the majority of the 200 s before the beginning of the post tilt phase. HbT showed only small time intervals where there was a significant difference. AOI patients cannot be grouped and averaged as the various AOI diagnoses (VS, POTS, OH, OHT) vary in BP and HR response. Therefore, to examine the hemodynamic responses more carefully, we separately investigated the AOI groups.

**Figure 5:**
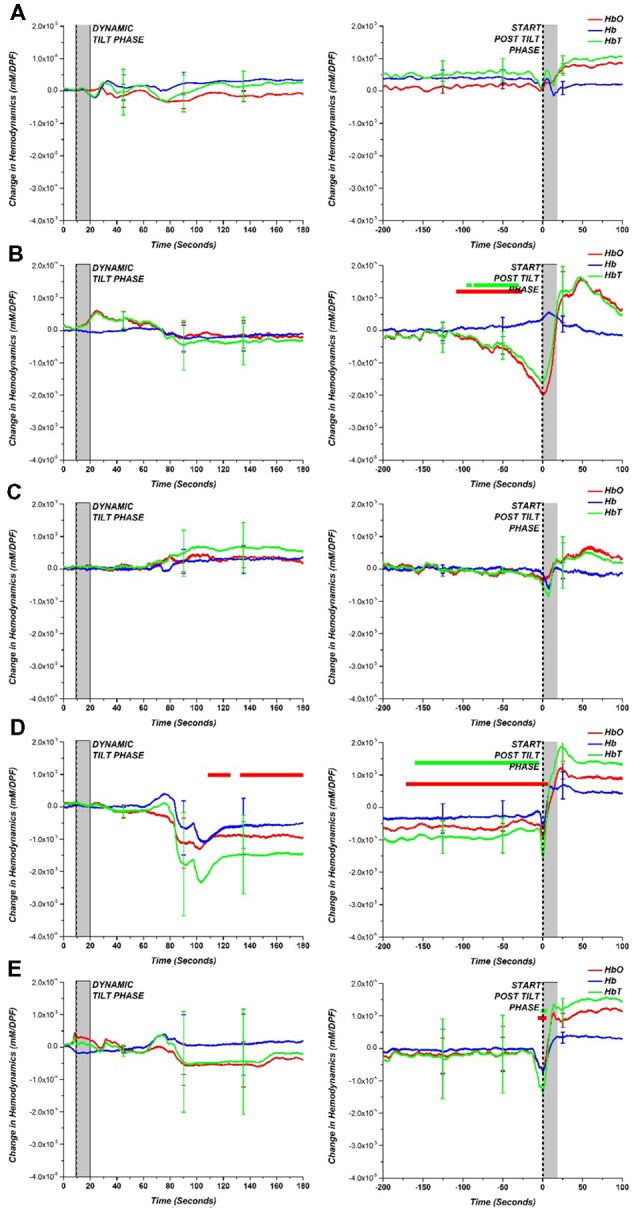
**(A)** HC compared to various tilt table diagnoses: **(B)** VS, **(C)** POTS, **(D)** OH and **(E)** OHT for dynamic tilt to static tilt phase (left) and the end of static tilt to post tilt phase (right). The gray bar marks the approximate mechanical tilting time (approximately 20 s, depending on weight). Statistically significant difference (*p* < 0.05) between tilt table diagnoses and HC is denoted with a dot for the respective hemodynamic response (HbO—red; Hb—blue; HbT—green).

Figure 5 shows the group average and a comparison between each of the AOI diagnoses results and the HC results obtained during the dynamic to static tilt phase and end of the static tilt to post tilt phase. This figure presents hemodynamic trends in the AOI diagnoses. Similar to Figure 4, the statistically significant differences between the AOI group and HC at each data point are marked with a dot matching the appropriate hemodynamic change (HbO-red; Hb-blue; HbT-green). Note that the limited number of subjects in the AOI diagnoses (i.e., POTS *n* = 2) may skew the results of the statistical tests, but trends in the NIRS data were present.

By separating the AOI groups, the OH group exhibited a large drop in HbO, Hb, and HbT concentration but only HbO was statistically different from HCs. Although consisting of only two patients, the POTS group showed a large error at the beginning of the static tilt phase, which may be due to the large increase in HR influencing the NIRS data. For the other groups, the largest difference between HCs can be seen at the end of the static tilt phase. VS showed a large drop in HbO and HbT, which may be attributed to syncope. The OH patient group did not show any recovery towards baseline levels for HbO and HbT by the end of the static tilt phase. OHT showed a large standard error within the patient group and demonstrated a large drop in HbO and HbT at the start of the post tilt phase.

**Figure 6:**
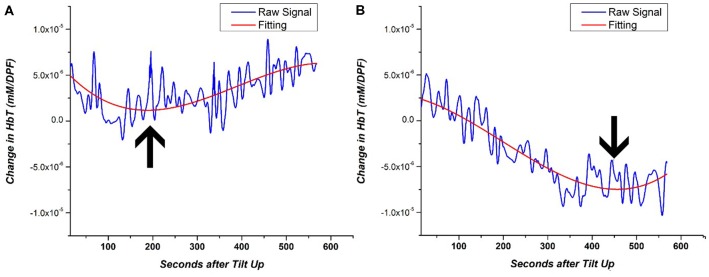
Example of 3rd order polynomial fitting for the HbT signal of **(A)** HC and **(B)** OI patient. The inflection point of the fitting line is denoted by the arrow. The inflection point during the HUT static period of the HUT is representative of the return to cerebral homeostasis.

As mentioned previously, the length of the static tilt phase is dependent on BP recovery or observation of OI symptoms. Cerebral perfusion ideally occurs during the static tilt phase when homeostasis is reestablished following postural changes. Rather than looking at time series changes of NIRS data that can fluctuate in time, we investigated a NIRS-based metric to identify when the cerebral perfusion hemodynamics begins to recover back to baseline. This metric is based on the inflection point of a polynomial line. First, we fitted each subject’s static phase HbT signal with a 3rd order polynomial line. The 3rd order polynomial fitting has previously been used to accurately fit and classify the NIRS signal (Thanh Hai et al., 2013). HbT was fitted as it is the best representation of blood volume changes and cerebral perfusion recovery in HUT. We then identified the inflection point of each subject’s NIRS-based polynomial fitting line to identify when the signal trends back to baseline. In the case of multiple inflection points, the curve that showed the greatest gradient value just prior to inflection was chosen. A visual depiction of how this NIRS metric quantifies perfusion is shown in Figure 6 for two subjects, a healthy subject, and an OI patient. By fitting the noisy time series data, we can clearly see that the healthy subject’s HbT concentration change leveled off approximately 200 s after tilting up. The OI patient’s blood volume continued to fall for approximately 450 s, then HbT began to trend toward baseline. The polynomial fitting is a method used for generally evaluating the delay in cerebral perfusion in OI patients using NIRS data.

**Figure 7:**
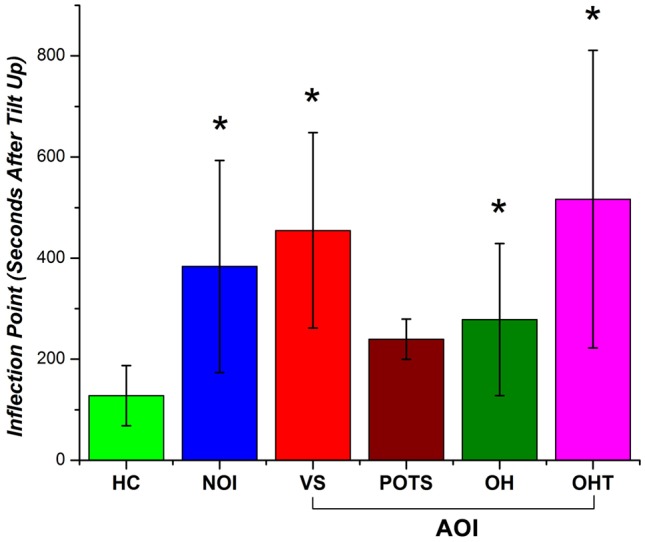
Inflection point occurrence of HbT changes as the number of seconds after tilt up (with standard error bars) for HC, NOI, and AOI (VS, POTS, OH, OHT). A statistical significant difference (*p*-value < 0.05) between HC and patient group is denoted by an asterisk (*).

The inflection point was calculated for each subject’s fitting line and recorded in terms of the number of seconds after the tilt up marker and was used as a metric to quantify the return of cerebral perfusion. Figure 7 shows the number of seconds after tilt up for the different patient groups. The mean (and standard error; SE) for the HCs was 127.8 ± 59.6 s while that of the NOI group was 383.5 ± 210.0 s. The difference was statistically significant (*p* = 0.004). The mean fir AOI group was divided according to the individual diagnoses: VS (mean, SE: 454.9 ± 193.5 s), POTS (mean, SE: 239.5 ± 39.7 s), OH (mean, SE: 278.4 ± 150.8 s), and OHT (mean, SE: 516.5 ± 294.5 s). We ran a two-tailed test to see if there was a difference in the sample mean between various groups. The AOI groups’ *p*-values compared to HCs were VS (*p* = 0.003), POTS (*p* = 0.13), OH (*p* = 0.031), and OHT (*p* = 0.007). There was no statistically significant difference either among the AOI groups or between the AOI and the NOI groups. Although there is a limited number of subjects within each group, these results show that HC NIRS data inflected much earlier than in the other group. The difference shown in Figure 7 between HC and all OI groups (NOI, AOI) demonstrate the potential of using the inflection point of HbT changes as an NIRS-based metric for differentiating between healthy subjects and OI patients.

## Discussion and Conclusion

Our study demonstrates that additional NIRS measurements during HUT can help detect OI symptoms in suspected OI patients. Although this study is limited due to the small number of patients in the subgroups, we were able to present trends that show the potential for enhanced OI monitoring using NIRS. By monitoring NIRS data, we observed trends that distinguished HC, NOI and AOI patients based on hemodynamic changes and a NIRS-based metric to quantify the return of cerebral perfusion after orthostatic challenges. Traditionally, the diagnosis of OI using HUT is performed only with BP and HR measurements. With this traditional method, NOI patients showed normal HUT results (i.e., recovery of BP and HR) and the diagnosis of OI could not be established. However, using NIRS during HUT showed that the NOI’s HbO and HbT cerebral changes may not return to baseline levels by the end of static tilt phase. In addition, both the NOI and AOI groups showed a delay in HbT signal inflection during static tilting, with the idea that cerebral blood volume is affected by varied cerebral perfusion pressure according to the cerebral autoregulation mechanism. Thus, a delayed HbT signal inflection in NOI and AOI patients may indicate a delay in the return to cerebral homeostasis compared to HCs. In other words, adding NIRS to the HUT can help detect OI symptoms better than traditional methods that use only the HUT to diagnose OI patients.

We have also shown the possibility of NIRS to differentiate between AOI diagnoses (VS, POTS, OH, and OHT) by providing characteristic signal patterns during HUT. However, our analysis is limited due to the small sample size within each group. For the AOI groups, we selected patients who showed the closest match to the clinical definition of the diagnoses. This gives us confidence that our selected subjects represent the typical BP, HR, and NIRS response for AOI groups. Table 1 shows that the median ages of AOI groups were in many cases lower than HCs. It has been shown that the age at onset of syncope is most common in the 10–30 age group, and it is known that the incidence of syncope increases rapidly after 65 years of age (Sheldon et al., 2006). Our subjects’ results seem to reflect this tendency with the collected BP/HR and NIRS data for these patient groups. This supports our intent to recruit the subjects who best represented each clinical diagnosis, even though the median age may deviate. Recruiting enough patients to show statistical significance based on this criteria is a difficult and time consuming process which would require a much larger OI patient pool. Further recruitment of patients for all groups will strengthen our statistical results for a more definite conclusion regarding cerebral perfusion during HUT as monitored by NIRS.

AOI group definition is beyond the scope of this project, however, our current patient groups did exhibit trends that coincide with BP and HR characteristics for the specific diagnosis. The VS subjects’ drop in cerebral blood volume and blood oxygenation correlated well with the drop in BP and HR. Additionally, there was a slight increase in Hb during the start of the post tilt phase which correlates with previous observations of cerebral deoxygenation during syncope (Bachus et al., 2018). A gradual decrease in HbO and HbT was distinctly observed before the syncope. This suggests that NIRS can be used to predict the onset of VS before clear cardio-circulatory symptoms arise. POTS patients showed high temporal fluctuation between subjects, particularly at the beginning of the static tilt phase. The large temporal fluctuation for the subjects is thought to be induced by the elevated HR in these patients. The analysis is limited by the small POTS sample size, so it is possible that POTS characteristics, not seen in VS, may not show any distinctive features in NIRS cerebral hemodynamics. The OH patients demonstrated a large drop in HbO, Hb, and HbT concentration approximately 100 s from the start of the static tilt phase, and there was no observable recovery back to baseline by the end of that phase. These results indicate that our OH patients showed a lack of recovery of cerebral hemodynamics during the entire static tilt phase of the HUT, which correlates well with the lack of recovery in BP data observed for OH symptoms. OHT patients showed a large amount of variation between subjects; therefore, it was difficult to identify clear trends in the NIRS signal. There is currently no widely agreed definition for clinical OHT, with the current definition being operational within the context of particular studies. In addition, the underlying pathophysiology is poorly understood. Further studies are needed to demonstrate whether OHT is just a manifestation of autonomic dysregulation or one of the leading causes of cerebrovascular diseases. Once more patients are recruited, further work can be carried out in order to characterize all AOI groups based on NIRS response.

Our ability to use NIRS to characterize HC, AOI, and NOI patients coincides with cerebral hemodynamics induced by HUT and lower body negative pressure. Syncope, a major OI symptom, demonstrates the importance of cerebral monitoring. It is defined as a transient loss of consciousness secondary to inadequate cerebral perfusion with oxygenated blood. Following standing, HUT, or lower body negative pressure, healthy people may occasionally experience syncope. Systemic BP and cerebral blood flow depend on gravity. In the standing position, body fluids shift towards the lower limbs. This leads to decreased venous return and lower cardiac output, inducing both the baroreceptor reflex and vestibulo-ANS to work in an attempt to prevent syncope. When BP changes occurs, the baroreceptor reflex adjusts HR and vascular resistance through sympathetic and vagal nerve activities. The vestibulo-ANS is stimulated by changes in gravity and is induced before BP changes. If these ANSs do not work efficiently, OH can occur (Rowell, 1993a,b; Arthur and Kaye, 2000; Nishimura and Yamasaki, 2018). In other words, a very rapid correction of plasma volume after standing may indicate that the body has an efficient fluid transfer system (Lundvall and Bjerkhoel, 1995). During HUT, the peripheral BP increases in caudal regions and causes hemoconcentration, which moves the plasma to the interstitial space and increases the intravascular osmotic pressure. This is one of the stimulators of vasopressin, which is important for maintenance of BP and blood flow during long-term HUT (Arthur and Kaye, 2000). The increase in epinephrine in response to orthostatic stress could stimulate venoconstriction, cardiac contractility, and peripheral vasodilation, through preferential stimulation of peripheral beta2-receptors. This leads to hemodilution caused by the influx of plasma into the vasculature. The cardiovascular effects of hormone imbalances and the increased rate of venous pooling may lead to syncope (Evans et al., 2001). Therefore inadequate cerebral perfusion may point to the body’s inability to properly compensate for the aforementioned body fluid shifts.

The study has some limitations that can be addressed in the future. Further investigation can be performed on the extent of regional dependency for cerebral perfusion (Figure 2). Although we showed that HCs have the highest number of channels with strongly positive correlation, all groups had channels with strong correlation in the center of the probe, which may be attributed to the high blood volume changes from the anterior and middle cerebral artery (Reinhard et al., 2014). Interestingly, the number of strongly positive channels were the lowest for the NOI group, which we believe may indicate why OI is difficult to diagnose in this group using traditional BP/HR measurements. The AOI group regional analysis was averaged with all diagnoses included; however, performing correlation analysis on each subgroup with more subjects added may reveal more about their regional dependency. Tomographic reconstruction of hemodynamic changes is a potential solution for revealing depth and spatial dependency. For the purpose of this study, we aimed to show the general trends of NIRS changes per group, and averaged all channels to remove the influence of noise in individual channels.

Also, since this study focuses on cerebral hemodynamic changes during posture changes, motion artifact correction is a critical issue. We used a wavelet filtering method which has been shown to be effective in removing motion artifacts (Molavi and Dumont, 2012); however, additional monitoring of position (i.e., gyroscope and accelerometer) (Strangman et al., 2018) could be used for more accurate motion artifact correction. There must be careful consideration for motion artifact correction during HUT as not to mask blood volume changes associated with cerebral perfusion rather than just the tilting procedure.

The effect of superficial hemodynamic response in the NIRS signal should also be investigated. Commonly, short channel regression is used to separate superficial hemodynamic changes. Our current probe’s smallest SD distance is 15 mm, a distance that is too long to only monitor superficial hemodynamic changes (Brigadoi and Cooper, 2015). To ensure that we are sensitive to cerebral hemodynamic changes, we chose the 30 mm and 36 mm channels, which offer the best tradeoff between gray matter sensitivity and noise level (Strangman et al., 2013). Nevertheless, the number of channels used in this study were numerous and monitored the entire prefrontal area for a detailed characterization of hemodynamic changes during the HUT.

Our study showed that the addition of NIRS to HUT allows better detection of OI symptoms compared to traditional methods in diagnosing patients with OI. More research in the future will enable the NIRS response to be used as an indicator of cerebral perfusion during orthostatic challenges. The clinical implementation of NIRS for OI may reveal more about the underlying factors, specifically those related to cerebral perfusion.

## Data Availability

All datasets generated for this study are included in the manuscript.

## Author Contributions

B-JK and B-MK contributed to the conception and design of the study. YK, S-HP, ZV and N-JJ conducted the study. YK and N-JJ collected the data. ZV, YK, S-HP, B-JK, B-MK: analyzed the data. ZV, YK, S-HP, BJK, B-MK interpreted the results. YK, S-HP and ZV: drafted the manuscript. YK, S-HP, ZV, B-JK and B-MK: approved the manuscript.

## Conflict of Interest Statement

The authors declare that the research was conducted in the absence of any commercial or financial relationships that could be construed as a potential conflict of interest.
